# Assessment of the potential shifts in the phenological development of representative spring plant species in Slovenia until the end of the 21st century using a model-based approach

**DOI:** 10.1007/s00484-026-03143-2

**Published:** 2026-03-23

**Authors:** Gal Oblišar, Gregor Gregorič, Andreja Sušnik, Marko Puškarić, Urša Vilhar

**Affiliations:** 1 Department of Forest Ecology, Slovenian Forestry Institute, Večna Pot 2, Ljubljana, 1000 Slovenia; 2 Department of Meteorological Support to Agriculture, Meteorology, Hydrology and Oceanography Office, Slovenian Environment Agency, Vojkova 1b, Ljubljana, 1000 Slovenia

**Keywords:** Plant phenology, Phenological model, Elevation dependence, Climate change

## Abstract

To assess the changes in the spring phenology in the future with particular emphasis on the elevation dependence of phenophase onset, a climate-driven phenological model was developed based on the spring indices methodology. Our study investigates both current and projected changes in the timing of flowering onset for common hazel (*Corylus avellana*), dandelion (*Taraxacum officinale*), and common lilac (*Syringa vulgaris*). We compiled comprehensive climate data and phenological records from 46 phenological stations of the National Phenological Network of the Slovenian Environment Agency for the period 1971–2020. In addition, we used climate projection data for the 21st century under two climate scenarios to evaluate potential future shifts in the onset of the selected phenophases. Specifically, we examined whether the agreement between model predictions and observed records varies with elevation during the reference period (1981–2010) and whether this relationship changes across three future climate periods: 2011–2040, 2041–2070, and 2071–2100. Model results indicate that spring phenophases are expected to occur earlier in Slovenia by the end of the 21st century, consistent with the projected increase in air temperatures. Moreover, the advancement in spring phenology will be more pronounced at higher elevations.

## Introduction

Phenology is the study of the periodically recurring patterns and behaviour of biological events, such as flowering and leaf unfolding in plants (Lieth 1974). In the context of climate change, phenology plays a crucial role in understanding ecosystem dynamics and biodiversity under changing environmental conditions. As temperatures rise and climate patterns shift due to global climate change, these phenological events are occurring earlier in many regions, which has significant implications for ecological interactions and agricultural practises (Parmesan and Yohe 2003; Menzel et al. 2006; Fu et al. 2015; Cui and Shi 2021). Advanced flowering times, pollinator migrations and breeding schedules shift established ecological relationships and can lead to mismatches that threaten the survival of species (Cleland et al. 2007; Forrest and Thomson 2011; Kudo and Ida 2013). Changes in plant phenology can also feedback to the climate system by influencing the exchange of water and energy between terrestrial ecosystems and the atmosphere (Richardson et al. 2013). Minimum and maximum air temperatures and the photoperiod play a crucial role in the phenological phases of plant flowering and leaf unfolding in spring (Schwartz et al. 2006). In recent decades, the occurrence of spring phenophases in the various species has become increasingly synchronised. The temporal differences between the onset of the growing season for different species at regional level (Wang et al. 2016) and along elevation gradients (Vitasse et al. 2018) are decreasing.

In recent years, phenology has evolved from an empirical topic of observing and recording the timing of some important annual natural events for selected species to a comprehensive scientific field that includes extended observations, experiments and modelling (Schwartz et al. 2006; Vilhar et al. 2018; Noumanovi et al. 2021). For phenological modelling of the interactions between ecosystems and the climate system, improved knowledge of phenological changes, their main drivers and the impact on the ecosystem is essential (Liu et al. 2019). Ground-based observations can accurately capture the timing of phenological events for specific sites and species. Networks of long‐term ground‐based phenological observations, such as those provided by national meteorological services (Vliet et al. 2003; Menzel et al. 2006; Aono and Kazui 2008) in accordance with World Meteorological Organization guidelines (Koch et al. 2007) or those conducted by forest research institutes following the harmonized guidelines of the International Co-operative Programme on Assessment and Monitoring of Air Pollution Effects on Forests (ICP Forests) (Vilhar et al. 2013) are particularly useful to investigate phenological variations over a large geographical area and their potential changes in response to climate change (Cleland et al. 2007). Given the increasing concern about climate change and its potential impacts, the establishment of international phenological networks has facilitated collaboration in large‐scale and standardized phenological data collection and sharing (Templ et al. 2018). More recently, the development of smartphones, automated cameras and other communication technologies has taken ground-based phenology monitoring by citizen science to a new level, significantly expanding the coverage of phenological events over a large area and for many more species (Dickinson et al. 2012; Hufkens et al. 2019). Slovenia has a very dense network of phenological stations with good coverage of the diverse Slovenian terrain. Phenological observations in Slovenia were organized in 1951 as part of the national phenological network within the framework of the agrometeorological service of the Hydrometeorological Institute of the Republic Slovenia in the former Yugoslavia. After 2001, phenological observations became a regular activity of the Department of Agrometeorology of the Environment Agency of the Republic of Slovenia. Initially, the observations were carried out at 30 phenological stations, later the number increased to over 200 stations in 1980. Currently, there are still 46 active phenological stations evenly distributed throughout the country at elevations ranging from 55 to 1050 m a.s.l. and with different site and climate characteristics. The number of phenological stations has declined because suitable observers are increasingly difficult to recruit in less populated areas, resulting in many contemporary phenological observations being integrated with nearby meteorological stations. In a global comparison, the Slovenian network of phenological observations stands out as one of the more consistently and continuously maintained phenological monitoring (Žust 2015).

Changes in plant phenophases in spring can have profound effects on the ecosystem dynamics, and due to the rapid climate changes in recent decades, predictions of these changes are becoming increasingly important. Since it is not possible to rely solely on long-term phenological observations to assess temporal phenological changes, models have been developed that simulate the onset of selected phenophases. These models, driven by daily maximum and minimum air temperatures, provide a biologically relevant approach to track phenological changes over larger spatial and temporal scales, using the availability of meteorological data. In contrast to raw meteorological data such as monthly or seasonal average air temperatures, models offer higher precision as they capture pheno-climatic processes on a daily to weekly scale that trigger critical events such as plant leafing and flowering that determine ecosystem dynamics. When a phenological event is triggered by a short period of extreme temperatures, this can be inadequately represented in general metrics such as monthly or seasonal average temperature (Schwartz et al. 2006; Gerst et al. 2020).

The aim of the present study was to assess the changes in the spring phenology in the future, focusing on the elevation dependence of phenophase occurrence. For this purpose, we developed a climate-driven phenological model based on the methodology of the American Spring Index using air temperature and spring phenology of the common hazel (*Corylus avellana*), dandelion (*Taraxacum officinale*) and common lilac (*Syringa vulgaris*). We used daily maximum and minimum air temperatures and phenological data on selected plant species and phenophases collected by at 46 phenological stations of the Slovenian Environment Agency for the period 1971–2020. Subsequently, we modelled the occurrence of selected phenophases until the end of the 21st century using the climate projections data of the EURO-CORDEX dataset for the RCP4.5 and RCP8.5 climate scenarios.

## Materials and methods

### Phenological data

**Fig. 1:**
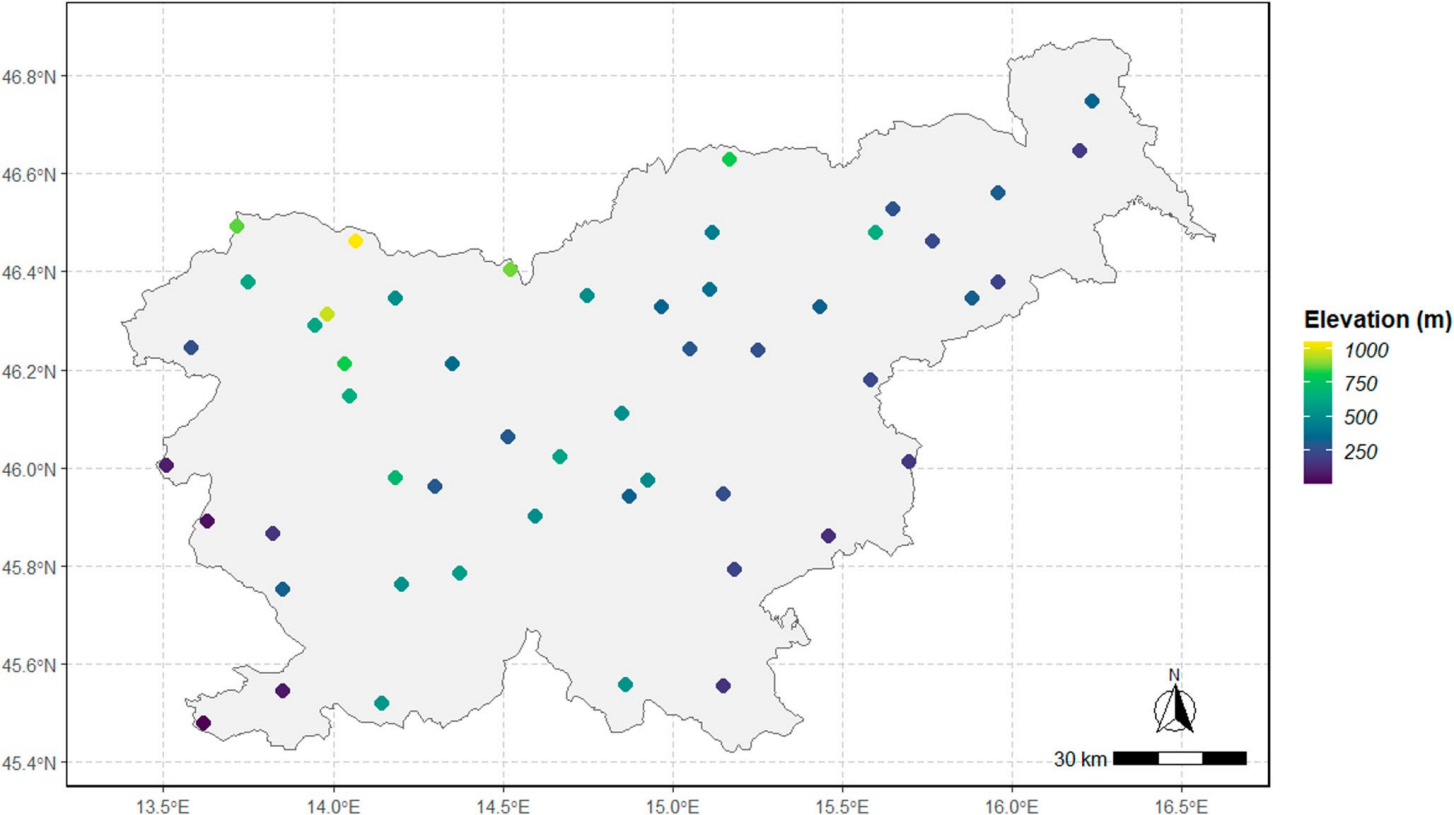
Locations with colour-coded elevation (m a.s.l.) of phenological stations included in this study

For the development and verification of the climate-driven phenological model, we selected 46 phenological stations of the national phenological network of the Slovenian Environment Agency with homogeneous and continuous data series evenly distributed across Slovenia (Fig. 1). Slovenia is characterised by relatively large gradients of climatic factors due to its location between the Alps, the Mediterranean and continental Europe (Čufar et al. 2012), and consequently a wide variety of habitats can be found in the country, from lowlands to high mountains (Kermavnar and Kutnar 2020).

The plant species and phenological phases included in the model were selected based on several criteria: (i) ease of identification and observation, (ii) average timing of occurrence to cover the entire spring period, (iii) wide distribution of the species across the country. Phenological stations were selected based on the homogeneity of their record for the selected phenophases and the presence of at least one continuous data series of 10 years or more during the period 1971–2020.

The selected plant species and phenological phases were the onset the male catkins flowering of the common hazel (*Corylus avellana*), the onset of flowering of the dandelion (*Taraxacum officinale*) and the onset of flowering of the common lilac (*Syringa vulgaris*).

Phenological observations at each phenological station were done according to the national guidelines and supervised by national phenological coordinator (Žust 2015), following the World Meteorological Organisation (WMO) guidelines for phenological observations (Koch et al. 2007). Observations were carried out daily. The onset of common hazel flowering was defined as stage when the two-part yellow anthers become visible on the elongated catkins, and the yellow pollen began to shed. This phase corresponds to Biologische Bundesanstalt, Bundessortenamt und Chemische Industrie code 60 (BBCH60). The onset of dandelion flowering was recorded when some fully developed and open flowers could be seen in the observed meadow (BBCH60). For common lilac, flowering was recorded when the first flowers opened at the lower edge of the first inflorescences and with two stamens visible per flower (BBCH60).

### Climate data

The meteorological data used were daily minimum and maximum air temperatures for the period 1971–2020, obtained from raster datasets provided by Slovenian Environment Agency (SEA). The air temperature data were recalculated to the elevation and geographical position of the selected phenological station using the methodology described in Huld and Pascua (2015).

Climate projection data for the 21st century were obtained from the EURO-CORDEX dataset, the European branch of the CORDEX initiative: EURO-CORDEX provides an ensemble of climate simulations generated by multiple dynamical and empirical-statistical downscaling models forced by several global climate models of the Coupled Model Intercomparison Project Phase 5 (CMIP5) (Gobiet and Jacob 2011). We considered two climate change scenarios based on the IPCC methodology: RCP4.5 – a moderately optimistic scenario that assumes a total radiative forcing of 4.5 W m^− 2^ by 2100 and a CO_2_ equivalent of 630 ppm by 2100, and RCP8.5 – a pessimistic scenario that assumes a total radiative forcing of 8.5 W m^− 2^ by 2100 and a CO_2_ equivalent of 1313 ppm by 2100 (IPCC 2021).

Climate models contain systematic biases arising from factors such as limited horizontal and vertical resolution, simplified equations for some physical processes, numerical approximations, incomplete understanding of all climate dynamics. In this study, the climate projection data used were bias-corrected within OPS21 project (Bertalanič et al. 2018), which applied a tailored quantile mapping approach to daily model outputs for the period 1981–2100. The reference period for bias correction was period 1981–2005 which represent historical simulations. Bias correction was applied separately for each model grid cell and time step using a moving 61-day window and 100 quantile classes. This approach preserved inter-variable dependencies and long-term trends while effectively reducing systematic errors in climate model projections. The climate projection dataset represents six combinations of regional and global climate models, providing with daily meteorological variables for a period 1981–2100 downscaled to the location of each phenological station. For model verification, we used the dataset for the period 1981–2005 and compared simulated values with observed phenological data against historical simulations. The period 2005–2100 represents bias-corrected climate projection dataset. Results of these projections are presented in three thirty-year climate periods: 2011–2040, 2041–2070 and 2071–2100 as a deviation in the timing of onset of individual phenophase compared to the reference period 1981–2010. 

### Phenological model and statistical analyses

We developed a climate-driven phenological model to assess future changes in the onset of flowering in selected plant species using climate projection data. The model was trained on long-term observational records from selected phenological stations and implemented in the MATLAB programming environment. It is based on the methodology of the American Spring Index, which incorporates the combined effects of air temperature and photoperiod. The American Spring Phenological Index model consists of two models that represent the average response of three reference species and estimate the “onset of spring” as either the first leaves emergence or the first flowering at a given location. These models were originally developed using observations of the first leaf and flowering phases of the *Syringa × chinensis* “*Red Rothomagensis”* and two honeysuckle clones (*Lonicera tatarica* “*Arnold Red*” and *Lonicera korolkowii Stapf)* (Schwartz et al. 2006, 2013; Ault et al. 2015; Rosemartin et al. 2015). To investigate the elevation dependence of phenophase occurrence, model results for the selected phenophases were analysed across the elevational gradient of the phenological stations. Model outputs represent the onset of the selected phenological phase expressed in Julian days (day of the year - DOY). Model performance was evaluated for the period 1981–2005. Relationships between observed and simulated phenophases were quantified using the Spearman’s correlation test. Model fit was further assessed with the coefficient of determination (R²), which indicates the proportion of variance in observed values explained by the model (Rodgers and Nicewander 1988). In addition, we calculated the root mean square error (RMSE), defined as the square root of the mean squared deviation between predicted estimated and observed values (Both et al. 2009). All statistical analyses were conducted in the programme R, version 4.2.3, with R packages: dplyr, data.table, stringr, lubridate, sp, tidyverse, broom, ggplot2 (R Development Core Team 2024). 

## Results

The climate-driven phenological model for the period 1971–2020.

During the period 1971–2020, the onset of common hazel male catkins flowering occurred on average at DOY 51 at all selected phenological stations, with observations ranging from DOY 29 at the Portorož station (2 m a.s.l.) to DOY 74 at the Planina pod Golico station (1050 m a.s.l.). The long-term trend indicated an advancement of 3.77 days per decade (R^2^ = 0.13) across all phenological stations.

Dandelion flowering onset was observed on average at DOY 100, ranging from DOY 71 at Portorož (2 m a.s.l.) to DOY 124 at Zgornje Jezersko (879 m a.s.l.). The mean trend was an advancement of 2.19 days per decade (R² = 0.15) across all phenological stations.

The onset of flowering of common lilac was observed on average on the DOY 120, ranging from DOY 103 at the Portorož station (2 m a.s.l.) to DOY 144 at the Planina pod Golico station (1050 m a.s.l.). The corresponding long-term trend showed an advancement of 3.45 days per decade (R² = 0.43).

**Fig. 2:**
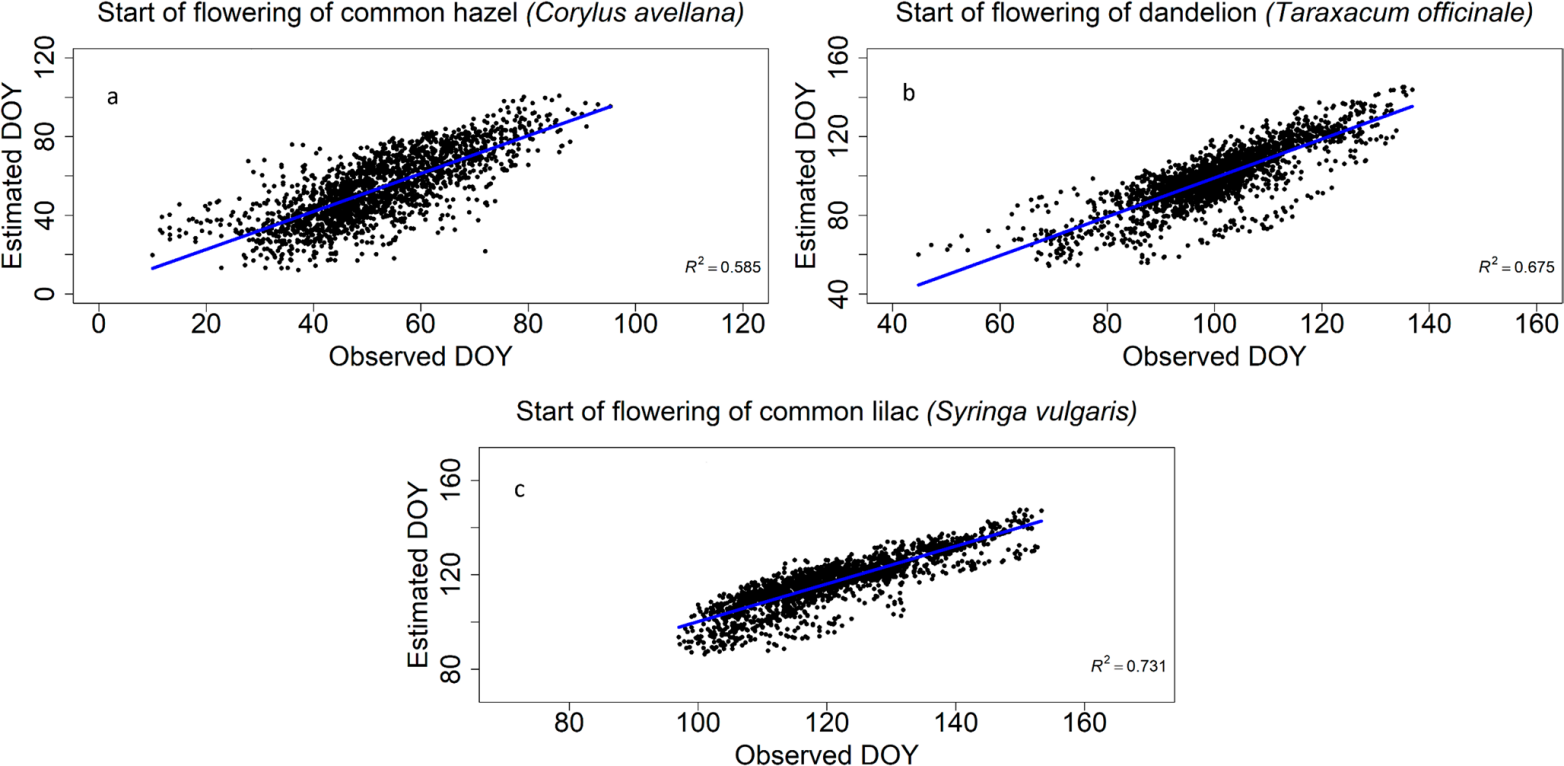
Scatter plots of observed versus modelled day of year (DOY) of phenological phases: onset of flowering of (**a**) male catkins of the common hazel, (**b**) onset of flowering of dandelion, and the (**c**) onset of flowering of common lilac across all phenological stations in Slovenia for the period 1971–2020. Blue lines indicate regression fits

The developed climate-driven phenological model demonstrated a good agreement between simulated and observed spring phenophases across all selected phenological stations. Model performance was slightly reduced at coastal stations and at higher-elevation sites in the Alps. The highest and statistically significant agreement was obtained for the onset of dandelion flowering (δ_avr_ = 0.86; *p* < 0.001), RMSE = 8.80, R^2^ = 0.68), followed by the onset of common lilac flowering (δ_avr_ = 0.80; *p* < 0.001), RMSE = 7.19, R^2^ = 0.73) and the onset of flowering of common hazel male catkins (δ_avr_ = 0.76; *p* < 0.001), RMSE = 11.05, R^2^ = 0.59) (Fig. 2; Table 1).

**Table 1:** Average day of the year (DOY) of observed phenophases: onset of flowering of male Catkins of the common Hazel (*Corylus avellana*), onset of flowering of dandelion (*Taraxacum officinale*) and common Lilac (*Syringa vulgaris*) across all selected phenological stations in Slovenia during the the period 1971–2020. Reported are spearman’s correlation coefficient (δ_avr_), significance levels (*p)*, coefficient of determination (R^2^), and root mean square errors (RMSE) comparing observed and modelled data, n denotes the total number of observations

	Mean_obs (DOY)_	δ_avr_	R^2^	RMSE	n
Onset of flowering of male catkins of the common hazel (*Corylus avellana*)	51.1	0.76***	0.59	11.06	2269
Onset of flowering of dandelion (*Taraxacum officinale*)	100	0.86***	0.68	8.80	2325
Onset of flowering of common lilac (*Syringa vulgaris*)	120.5	0.80***	0.73	7.19	2300

* p < 0.05, ** p < 0.01, *** p < 0.001

Agreement between observed and modelled spring phenology using climate projection data for the reference period 1981–2010.

The climate-driven model showed a high and statistically significant agreement between observed and simulated onset of common lilac flowering in 6-year moving averages using the EURO-CORDEX climate dataset for the reference period 1981–2005, under both climate scenarios RCP4.5 and RCP8.5 (δ_avr_ = 0.84; *p* < 0.001; RMSE_RCP4.5_ = 6.94, RMSE_RCP8.5_ = 6.99). Agreement was slightly lower for the onset of dandelion flowering (δ_avr_ = 0.80 (*p* < 0.001; RMSE_RCP4.5_ = 6.43) [RCP4.5] and δ_avr_ = 0.79 (*p* < 0.001; RMSE_RCP8.5_ =6.41) [RCP8.5]. The lowest agreement was observed for common hazel male catkins flowering with δ_avr_ = 0.55 (*p* < 0.001; RMSE_RCP4.5_ = 20.43) [RCP4.5] and δ_avr_ = 0.46 (*p* < 0.01; RMSE_RCP8.5_ =22.53) [RCP 8.5] (Table 2).

**Table 2:** Spearman’s corellation coefficient (δavr), significance levels (p) and root mean square errors (RMSE) between observed and simulated spring phenophases on two climate change climate scenarios RCP4.5 and RCP8.5 using EURO-CORDEX climate dataset for the reference period 1981–2005

	RCP 4.5		RCP 8.5	
	δavr	RMSE	δavr	RMSE
Onset of flowering ofmale catkins of thecommon hazel (*Corylus* *avellana*)	0.55***	20.43	0.46**	22.53
Onset of flowering ofdandelion (*Taraxacum* *officinale*)	0.80***	6.43	0.79***	6.41
Onset of flowering ofcommon lilac (*Syringa* *vulgaris*)	0.84***	6.94	0.84***	6.99

* p < 0.05, ** p < 0.01, *** p < 0.001

Modelled changes in spring phenology until the end of the 21st century.

**Fig. 3:**
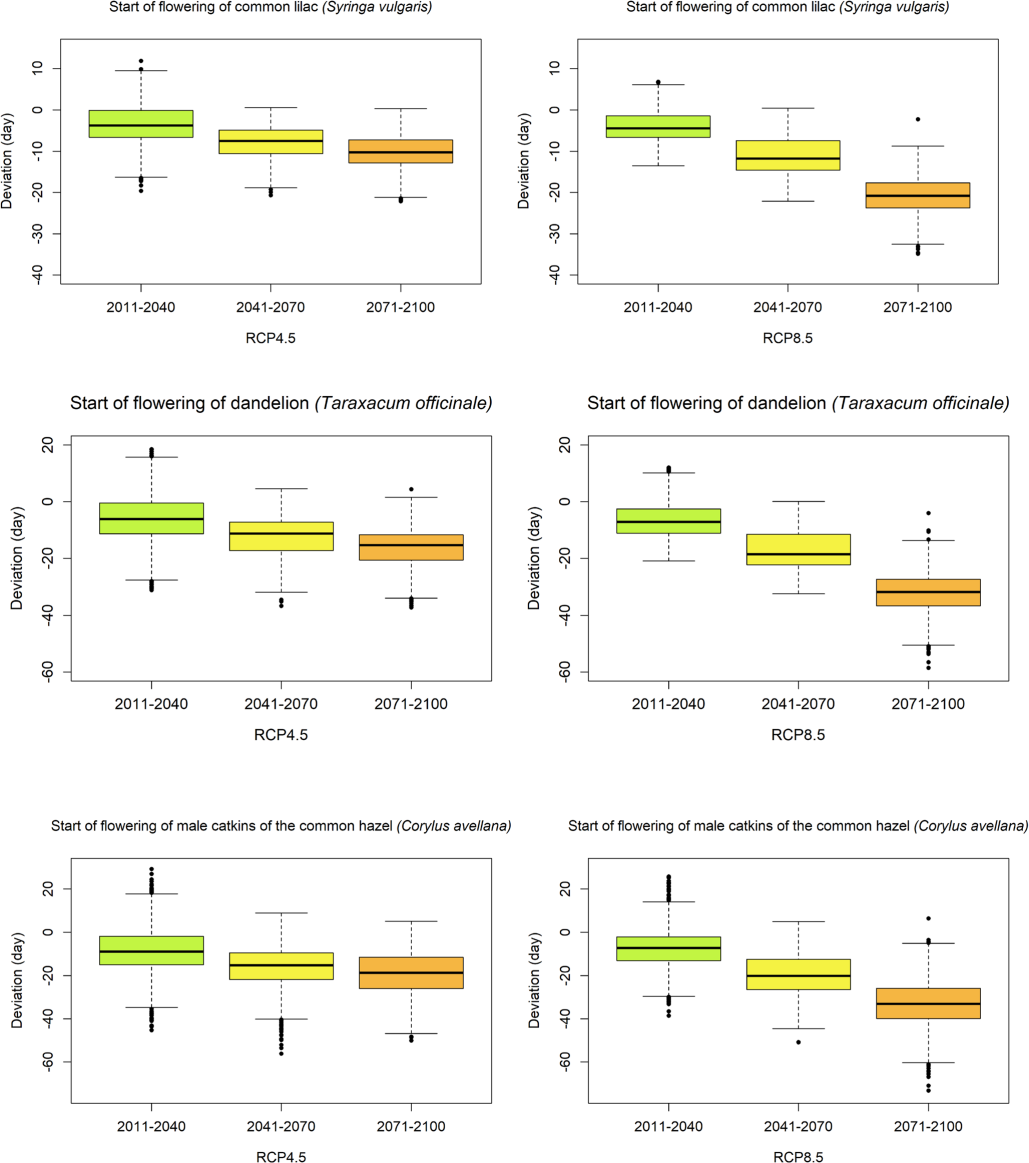
Average deviation of modelled phenophases onset under climate scenarios RCP4.5 (left panels) and RCP8.5 (right panels) for three future periods 2011–2040, 2041–2070 and 2071–2100. Deviations are calculated relative to average phenophase occurrence during period 1981–2010 across all selected phenological stations in Slovenia. Panels show the onset of flowering for common lilac (*Syringa vulgaris*) (first row), dandelion (*Taraxacum officinale*) (second row) and male catkin of the common hazel (*Corylus avellana*) (third row)

Climate-driven model projections indicate that all three studied spring phenophases in Slovenia are expected to occur earlier by the end of the 21st century, consistent with the projected rise in expected air temperatures (IPCC 2021). For common lilac, under RCP4.5 the onset of flowering is projected to advance by 4.0 days in 2011–2040 relative to 1981–2010, increasing to 7.5 days in 2041–2070 and 10.3 days in 2071–2100. Under RCP8.5, advances are slightly larger: 4.5 days (2011–2040), 11.8 days (2041–2070), and 20.8 days (2071–2100). The maximum modelled deviation in the last period 2071–2100 for the onset of common lilac flowering from the average for the period 1981–2010 was modelled at 22.1 days for the RCP4.5 climate scenario and 34.9 days for the RCP8.5 climate scenario. The climate-driven model predicts that the dandelion under RCP4.5 will onset flowering 6.1 days earlier in 2011–2040 relative to 1981–2010, increasing to 11.2 days 2041–2070 and 15.3 days in 2071–2100. Under RCP8.5, advances are even larger: 7.0 days (2011–2040), 18.5 days (2041–2070), and 31.8 days (2071–2100). The maximum deviation in the last period 2071–2100 for the onset of dandelion flowering from the 1981–2010 average was modelled at 37.2 days for the RCP4.5 climate scenario and 58.5 days for the RCP8.5 climate scenario. For common hazel, under RCP4.5 the onset of flowering of the male catkins is projected to advance by 7.2 days in 2011–2040 relative to 1981–2010, increasing to 15.2 days in 2041–2070 and 18.7 days in 2071–2100. Under RCP8.5 advances are larger 8.9 days (2011–2040), 20.1 days (2041–2070), and 33.1 days (2071–2100). The maximum deviation in the last period 2071–2100 for the onset of flowering of the male catkins of the common hazel from the average of the period 1981–2010 was modelled at 50.1 days for the climate scenario RCP4.5 and 73.2 days for the climate scenario RCP8.5 (Fig. 3).

Elevation dependence of spring phenology until the end of the 21st century.

**Fig. 4:**
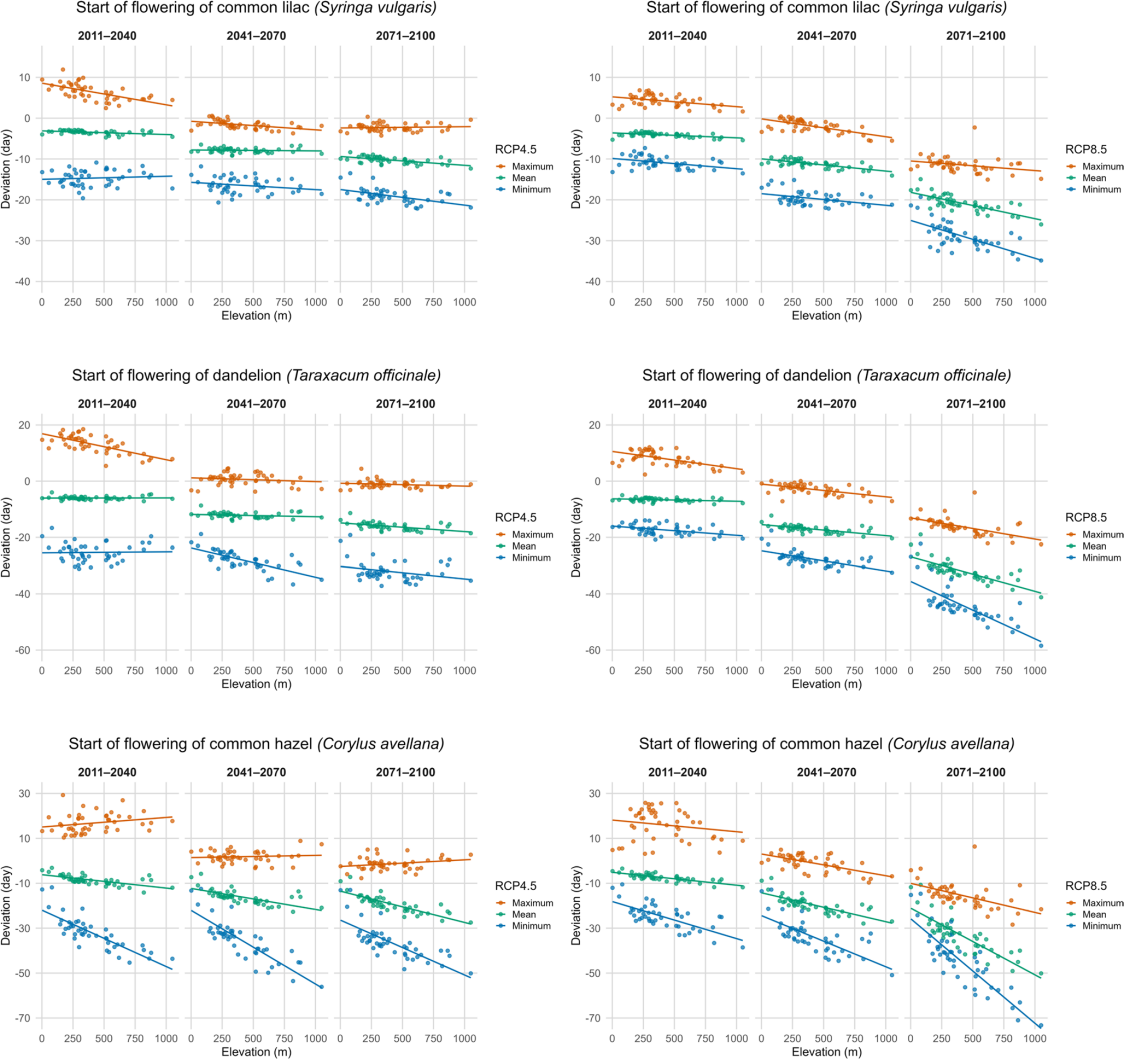
Summary statistics (maximum, mean and minimum deviation) of modelled phenophase onset under climate scenarios RCP4.5 (left panels) and RCP8.5 (right panels) shown along a continuous elevation gradient. Deviations are calculated relative to the average phenophase onset during the reference period 1981–2010 across all selected phenological stations in Slovenia. Each panel presents the relationship between elevation and phenophase deviation for three future climate periods (2011–2040, 2041–2070, 2071–2100), including fitted linear trends. Rows correspond to the onset of flowering of common lilac (*Syringa vulgaris*) (first row), dandelion (*Taraxacum officinale*) (second row) and male catkins of common hazel (*Corylus avellana*) (third row)

To assess the elevation dependence of spring phenology, model results for the selected phenophases were analysed across the elevational gradient of the phenological stations. Results are presented as deviation of selected future period from 1981 to 2010 (Fig. 4). The the magnitude of changes in selected phenophases varies with elevation. For the onset of flowering of common lilac, the results show a generally negative relationship between elevation and deviation of phenological development under both RCP4.5 and RCP8.5. In all periods (2011–2040, 2041–2070, 2071–2100), higher-elevation stations tend to exhibit stronger advancements in flowering (more negative deviations). The slope becomes steeper through time, particularly under RCP8.5, indicating that elevation increasingly amplifies the phenological response as warming intensifies. A similar pattern is observed for the onset of flowering of dandelion. Under RCP4.5, the elevation effect is relatively weak in the early period but becomes more pronounced by mid- and late century. Under RCP8.5, the elevation dependence is already visible in 2011–2040 and strengthens considerably by 2071–2100. Across all periods, stations situated at higher altitudes show earlier flowering relative to the period 1981–2010. For the flowering of male catkins of common hazel, elevation exhibits a clear and increasingly strong influence, especially under RCP8.5. While RCP4.5 shows a moderate negative trend with elevation, the late-century projections under RCP8.5 reveal a very steep relationship, with high-altitude stations advancing by more than 40 days relative to the period 1981–2010. This highlights a common hazel as particularly sensitive to temperature changes in a high-warming scenario. Overall, a consistent pattern across all species is that higher elevations are associated with greater advancements in spring phenophases, and this dependence strengthens both over time and with increasing climate forcings.

## Discussion

Plant phenology has been shown to be a very sensitive indicator of the effects of climate change (Menzel et al. 2006). This study investigates the potential shifts in spring phenology of the three most wide distributed plants in Slovenia under two different climate scenarios using a newly developed climate-driven phenological model. The model is based on the Spring Indices (SI) model, which is a set of complex phenological models that have been successfully applied to assess variations and trends in spring onset in temperate regions of the Northern Hemisphere (Schwartz et al. 2013). Previous studies shown that one-phase models perform comparably to biphasic models for most species (Vitasse et al. 2011) when tested phenological models using phenological observations of six European tree species collected over 2–3 years.

The advance of spring phenology as a result of air temperature increase was extensively documented and mostly showed a trend towards advancement, although variation in predicted phenology may reflect differences in study scale and models used (Fu et al. 2015; Asse et al. 2020; Wang et al. 2022; Zimmer et al. 2022). Insufficient chilling may limit phenological advancement in response to warming at lower elevations and/or lower latitudes (Fu et al. 2015), suggesting that future studies should consider additional phenophases and chilling requirements. The present study shows that the onset of male catkins flowering of the common hazel is highly dependent on climate conditions, in particular air temperature, resulting in considerable interannual variability. This phenophase exhibited the highest standard deviation among all investigated phenophases, which is consistent with previous findings (Piskornik et al. 2001; Taghavi et al. 2018); Črepinšek et al. (2012) who reported advancement of 7.0–8.8 days per 1 °C increase in air temperature. Our study has indicated that under RCP8.5 scenario with projected 3.0–5.1 °C increase in spring warming by the end of 21st century could advance flowering of common hazel for 8.17 days/ 1 °C. Bergant et al. (2001) reported that the flowering of dandelion (*Taraxacum officinale*) in the period 2020–2049 will advance for 10–11 days compared to the period 1960–1989 or 1.6–1.8 days per decade. Our simulations showed comparable results, under RCP4.5 scenario, where dandelion flowering advances by 6.1 days or 2.0 days/decade in the period 2011–2040; 11.2 days or 1.87 days/decade in the period 2041–2070 and 15.7 days or 1.7 days/decade in the period 2071–2100. Under RCP8.5, the corresponding advances are 2.36, 3.08 and 3.53 days/decade. These findings align with Hu et al. (2022), who reported that the mean trend of spring phenology of the 14 species for the period 2021–2099 will be − 1.30 days/decade (RCP4.5) and − 2.79 days/decade (RCP8.5). This is consistent with our results on different plants with comparable spring phenophase timing, with modelled average trend of -1.9 days/decade (RCP4.5) and in the − 2.68 days/decade (RCP8.5). The strongest response was observed for the onset of male catkins flowering of common hazel − 2.62 days/decade (RCP4.5); -3.14 days/decade (RCP8.5) and the weakest shift was recorded for onset of common lilac flowering of (-1.22 days/decade, RCP4.5; -1.92 days/decade, RCP8.5).

Our study showed that spring phenology will continue to advance at both moderate and high emission scenarios, with species specifics differences in sensitivity. It will be greater at higher elevations in the future, which is consistent with previous studies (Güsewell et al. 2017; Vitasse et al. 2018; Mei et al. 2021). Onset of flowering for all three studied species mostly have the negative relationship between elevation and phenophase timing which strengthens over 21st century. Overall, our study confirms that both climate and elevation exert significant influence on current and future spring phenology in the investigated species.

The projected advancements in spring phenophases, have important ecological implications. Earlier flowering can alter plant–pollinator interactions by shifting resource availability before key pollinator species become active, increasing the risk of phenological mismatches (Parmesan 2006). Such desynchronization may reduce pollination success, affect plant reproductive fitness, and influence pollinator population dynamics, especially for specialist species (Memmott et al. 2007). Changes in flowering timing may also reshape competitive relationships within plant communities, favouring early responding species and potentially altering biodiversity patterns (Forrest and Miller-Rushing 2010). In forest and grassland ecosystems, advanced spring development can modify the seasonal availability of nectar and pollen, influencing food-web dynamics and the timing of insect emergence (Thackeray et al. 2016). Overall, the elevation-dependent trends identified in this study suggest that climate change may increase the likelihood of temporal mismatches and cascading ecological effects, particularly in high-elevation environments where phenological shifts are strongest.

The climate-driven phenology model was constructed using data from phenological stations in different regions of Slovenia, however, given the pronounced climatic heterogeneity within relatively small geographic area, regional differences may have introduced bias. Future research should include additional datasets and improvements (regional characteristics, etc.) into the model to improve accuracy and reliability of phenological projections.

## Conclusions

This study demonstrates that rising air temperature advance the onset of spring phenology in selected plant species in Slovenia throughout 21st century. In all considered species, and climate scenarios the advancement of flowering is more pronounced at higher elevations, highlighting the interaction between temperature increase and topography. Overall, these results provide strong evidence that future warming will substantially alter spring phenology in Slovenia, with species-specific and elevation-dependent sensitivities. This underlines the importance of phenological monitoring and climate-informed modelling as essential tools for assessing ecological impacts and informing adaptation strategies under ongoing climate change.

## Data Availability

The data presented in this study are openly available in FigShare at 10.6084/m9.figshare.30233563.v1, reference number 30233563.

## References

[CR1] Aono Y, Kazui K (2008) Phenological data series of Cherry tree flowering in Kyoto, Japan, and its application to reconstruction of springtime temperatures since the 9th century. Int J Climatol 28(7):905–914. 10.1002/joc.1594

[CR2] Asse D, Randin CF, Bonhomme M, Delestrade A, Chuine I (2020) Process-based models outcompete correlative models in projecting spring phenology of trees in a future warmer climate. Agric for Meteorol 285:107931. 10.1016/j.agrformet.2020.107931

[CR3] Ault TR, Schwartz MD, Zurita-Milla R, Weltzin JF, Betancourt JL (2015) Trends and natural variability of spring onset in the coterminous united States as evaluated by a new gridded dataset of spring indices. J Clim 28:21: 8363–8378

[CR4] Bergant K, Kajfež-Bogataj L, Črepinšek Z (2001) Statistical downscaling of general-circulation-model- simulated average monthly air temperature to the beginning of flowering of the dandelion (Taraxacum officinale) in Slovenia. Int J Biometeorol 46(1):22–32. 10.1007/s00484-001-0114-y 10.1007/s00484-001-0114-y 11931095

[CR5] Bertalanič R, Dolinar M, Draksler A, Honzak L, Kobold M, Kozjek K, Lokošek N, Medved A, Vertačnik G, Vlahović Ž, Žust A (2018) Ocena podnebnih sprememb v Sloveniji do konca 21. stoletja: sintezno poročilo. Ljubljana: Ministrstvo za okolje in prostor, Agencija Republike Slovenije za okolje. [in Slovenian] https://meteo.arso.gov.si/uploads/probase/www/climate/text/sl/publications/OPS21_Porocilo.pdf

[CR6] Both C, Van Asch M, Bijlsma RG, Van Den Burg AB, Visser ME (2009) Climate change and unequal phenological changes across four trophic levels: constraints or adaptations? J Anim Ecol 78:73–83. 10.1111/j.1365-2656.2008.01458.x 18771506 10.1111/j.1365-2656.2008.01458.x

[CR7] Cleland EE, Chuine I, Menzel A, Mooney HA, Schwartz MD (2007) Shifting plant phenology in response to global change. Trends Ecol Evol 22(7):357–365 17478009 10.1016/j.tree.2007.04.003

[CR9] Črepinšek Z, Stampar F, Kajfež-Bogataj L, Solar A (2012) The response of Corylus Avellana L. phenology to rising temperature in north-eastern Slovenia. Int J Biometeorol 56(4):681–694 21786017 10.1007/s00484-011-0469-7

[CR10] Čufar K, De Luis M, Saz MA, Črepinšek Z, Kajfež-Bogataj L (2012) Temporal shifts in leaf phenology of beech (Fagus sylvatica) depend on elevation. Trees 26, 1091–1100 (2012). 10.1007/s00468-012-0686-7

[CR8] Cui L, Shi J (2021) Evaluation and comparison of growing season metrics in arid and semi-arid areas of Northern China under climate change. Ecol Indicat 121:107055. 10.1016/j.ecolind.2020.107055

[CR34] R Development Core Team (2024) R: A language and environment for statistical computing. R Foundation for Statistical Computing, Vienna, Austria. https://www.R-project.org/

[CR11] Dickinson JL, Shirk J, Bonter D, Bonney R, Crain RL, Martin J, Phillips T, Purcell K (2012) The current state of citizen science as a tool for ecological research and public engagement. Front Ecol Environ 10:291–297. 10.1890/110236

[CR12] Forrest J, Miller-Rushing AJ (2010) Toward a synthetic Understanding of the role of phenology in ecology and evolution. Philosophical Trans Royal Soc B 365:3101–3112 10.1098/rstb.2010.0145 PMC2981948 20819806

[CR13] Forrest J, Thomson JD (2011) An examination of the effects of climate change on the phenology of flowering plants and their pollinators. Glob Change Biol 17(2):1160–1172

[CR14] Fu YH, Zhao H, Piao S, Peaucelle M, Peng S, Zhou G, Ciais P, Huang M, Menzel A, Peñuelas J, Song Y, Vitasse Y, Zeng Z, Janssens IA (2015) Declining global warming effects on the phenology of spring leaf unfolding. Nature 526(7571):104–107. 10.1038/nature15402 26416746 10.1038/nature15402

[CR15] Gerst KL, Crimmins TM, Posthumus EE, Rosemartin AH, Schwartz MD (2020) How well do the spring indices predict phenological activity across plant species? International Journal of Biometeorology, 2020;64:1825–1837. 10.1007/s00484-020-01879-z 10.1007/s00484-020-01879-z 32107635

[CR16] Gobiet A, Jacob D (2011) The EURO-CORDEX-Initiative. WCRP Open Science Conference Denver, 24–28 Oct. 2011. Poster no. W108B

[CR17] Güsewell S, Furrer R, Gehrig R, Pietragalla B (2017) Changes in temperature sensitivity of spring phenology with recent climate warming in Switzerland are related to shifts of the preseason. Glob Change Biol 23(12):5189–5202. 10.1111/gcb.13781 10.1111/gcb.13781 28586135

[CR18] Hu Z, Wang H, Dai J, Ge Q, Lin S (2022) Stronger spring phenological advance in future warming scenarios for temperate species with a lower chilling sensitivity. Front Plant Sci 13:830573 35665167 10.3389/fpls.2022.830573 PMC9158521

[CR19] Hufkens K, Melaas EK, Mann ML, Foster T, Ceballos F, Robles M, Kramer B (2019) Monitoring crop phenology using a smart- phone based near‐surface remote sensing approach. Agric for Meteorol 265:327–337. 10.1016/j.agrformet.2018.11.002 agrformet.2018.11.002

[CR20] Huld T, Pascua I (2015) Spatial downscaling of 2-Meter air temperature using operational forecast data. Energies 8(4):2381–2411

[CR21] IPCC (2021) Climate change 2021: the physical science Basis. Contribution of working group I to the sixth assessment report of the intergovernmental panel on climate change. Cambridge University Press

[CR22] Kermavnar J, Kutnar L (2020) Patterns of Understory Community Assembly and Plant Trait-Environment Relationships in Temperate SE European Forests. Diversity 2020, 12, 91. 10.3390/d12030091

[CR23] Koch E, Bruns E, Chmielewski FM, Defila C, Lipa W, Menzel A (2007) Guidelines for Plant Phenological Observations. WMO-No. 1028, World Meteorological Organization, Geneva. Switzerland 2007

[CR24] Kudo G, Ida TY (2013) Early onset of spring increases the phenological mismatch between plants and pollinators. Ecology 94:2311–2320. 10.1890/12-2003.1 24358716 10.1890/12-2003.1

[CR25] Lieth H (1974) Phenology and Seasonality Modeling. Springer–Verlag, New York (1974)

[CR26] Liu Q, Piao S, Fu YH, Gao M, Peñuelas J, Janssens IA (2019) Climatic warming increases Spatial synchrony in spring vegetation phenology across the Northern hemisphere. Geophys Res Lett 46:1641–1650. 10.1029/2018GL081370

[CR27] Mei L, Bao G, Tong S, Yin S, Bao Y, Jiang K, Hong Y, Tuya A, Huang X (2021) Elevation-dependent response of spring phenology to climate and its legacy effect on vegetation growth in the mountains of Northwest Mongolia. Ecol Ind 126:107640

[CR28] Memmott J, Craze PG, Waser NM, Price MV (2007) Global warming and the disruption of plant–pollinator interactions. Ecol Lett 10:710–717 17594426 10.1111/j.1461-0248.2007.01061.x

[CR29] Menzel A, Sparks TH, Estrella N (2006) European spring phenology and climate change. Glob Change Biol 12(2):321–329

[CR30] Noumanovi KD, Oblišar G, Žust A, Vilhar U (2021) Empirical approach for modelling tree phenology in mixed forests using remote sensing. Remote Sens 2021(3015). 10.3390/rs13153015

[CR32] Parmesan C (2006) Ecological and evolutionary responses to recent climate change. Annu Rev Ecol Evol Syst 37:637–669

[CR31] Parmesan C, Yohe G (2003) A globally coherent fingerprint of climate change impacts across natural systems. Nature 421(6918):37–42 12511946 10.1038/nature01286

[CR33] Piskornik Z, Wyzgolik GM, Piskornik M (2001) Flowering of hazelnuts cultivars from different regions under the Climatic conditions of Southern Poland. Acta Hortic 556:529–535

[CR35] Richardson AD, Keenan TF, Migliavacca M, Ryu Y, Sonnentag O, Toomey M (2013) Climate change, phenology, and phenological control of vegetation feedback to the climate system. Agric for Meteorol 169:156–173. 10.1016/j.agrformet.2012.09.012

[CR36] Rodgers J, Nicewander WA (1988) Thirteen ways to look at the correlation coefficient. Am Stat 42(1):59–66. 10.1080/00031305.1988.10475524

[CR37] Rosemartin AH, Denny EG, Weltzin JF, Lee Marsh R, Wilson BE, Mehdipoor H, Zurita-Milla R, Schwartz MD (2015) Lilac and honeysuckle phenology data 1956–2014. Sci Data 2:1: 150038. 10.1038/sdata.2015.38 26306204 10.1038/sdata.2015.38 PMC4520215

[CR38] Schwartz MD, Ahas R, Zalan E (2006) Onset of spring starting earlier across the Northern hemisphere. Glob Change Biol 12(2):343–351

[CR39] Schwartz MD, Ault TR, Betancourt JL (2013) Spring onset variations and trends in the continental united states: past and regional assessment using temperature-based indices. Int J Climatol 33:13: 2917–2922

[CR40] Taghavi T, Dale A, Saxena P, Galic D, Rahemi A, Kelly J, Suarez E (2018) Flowering of hazelnut varieties and how it relates to temperature in Southern Ontario. Acta Hort 1226:131–136. 10.17660/ActaHortic.2018.1226.18

[CR41] Templ B, Koch E, Bolmgren K, Ungersböck M, Paul A, Scheifinger H, Rutishauser T, Busto M, Chmielewski FM, Hájková L, Hodzić S, Kaspar F, Pietragalla B, Romero-Fresneda R, Tolvanen A, Vučetič V, Zimmermann K, Žust A (2018) Pan European phenological database (PEP725): A single point of access for European data. Int J Biometeorol 62:1–5. 10.1007/s00484-018-1512-8 29455297 10.1007/s00484-018-1512-8

[CR42] Thackeray SJ, Henrys PA, Hemming D, Bell JR, Botham MS, Burthe S, Helaouet P, Johns DG, Jones ID, Leech DI, Mackay EB, Massimino D, Atkinson S, Bacon PJ, Brereton TM, Carvalho L, Clutton-Brock TH, Duck C, Edwards M, Elliott JM, Hall SJG, Harrington R, Pearce-Higgins JW, Høye TT, Kruuk LEB, Pemberton JM, Sparks TH, Thompson PM, White I, Winfield IJ, Wanless S (2016) Phenological sensitivity to climate across taxa and trophic levels. Nature, 2016;535(7611):241–245. 10.1038/nature18608 10.1038/nature18608 27362222

[CR44] Vilhar U, Beuker E, Mizunuma T, Skudnik M, Lebourgeois F, Soudani K, Wilkinson M (2013) Chap. 9. Tree Phenology. Forest Monitoring, Terrestrial Methods in Europe with Outlook to North America and Asia. M. Ferretti, R. Fischer. Amsterdam, Elsevier: 12: 169–182

[CR45] Vilhar U, De Groot M, Žust A, Skudnik M, Simončič P (2018) Predicting phenology of European Beech in forest habitats. iForest 11:41–47. 10.3832/ifor1820-010

[CR46] Vitasse Y, François C, Delpierre N, Dufrêne E, Kremer A, Chuine I, Delzon S (2011) Assessing the effects of climate change on the phenology of European temperate trees. Agric for Meteorol 151(7):969–980. 10.1016/j.agrformet.2011.03.003

[CR47] Vitasse Y, Signarbieux C, Fu YH (2018) Global warming leads to more uniform spring phenology across elevations. Proc Natl Acad Sci USA 115(5):1004–1008. 10.1073/pnas.1717342115 29279381 10.1073/pnas.1717342115 PMC5798366

[CR43] Vliet AJH, de Groot RS, Bellens Y, Braun P, Bruegger R, Bruns E, Clevers J, Estreguil C, Flechsig M, Jeanneret F, Maggi M, Martens P, Menne B, Menzel A, Sparks T (2003) The European phenology network. Int J Biometeorol 47:202–212. 10.1007/s00484-003-0174-2 12734744 10.1007/s00484-003-0174-2

[CR48] Wang C, Tang Y, Chen J (2016) Plant phenological synchrony increases under rapid within-spring warming. Sci Rep 6(1):25460. 10.1038/srep25460 27145698 10.1038/srep25460 PMC4857096

[CR49] Wang H, Lin S, Dai J, Ge Q (2022) Modeling the effect of adaptation to future climate change on spring phenological trend of European Beech (Fagus sylvatica L). Sci Total Environ 846:157540 35878847 10.1016/j.scitotenv.2022.157540

[CR50] Zimmer SN, Reeves MC, St. Peter JR, Hanberry BB (2022) Earlier green-up and senescence of temperate united States rangelands under future climate. Model Earth Syst Environ 8:5389–5405. https://doi-org

[CR51] Žust A (2015) Fenologija v sloveniji: Priročnik Za fenološka Opazovanja [Phenology in slovenia: manual for phenological observations]. Ministry of Environment, Slovenian Environment Agency, Ljubljana, Slovenia, p 104. [in Slovenian]

